# Supplementary material for: Inflammatory factors and risk of lung adenocarcinoma: a Mendelian randomization study mediated by blood metabolites
Source: Front Endocrinol (Lausanne). 2024 Aug 27;15:1446863. doi: 10.3389/fendo.2024.1446863 (PMC11384989; doi:10.3389/fendo.2024.1446863)
Supplement: Supplementary file 1 [file DataSheet1.zip › supplementary files/S2/GCST90200582/forest.pdf]

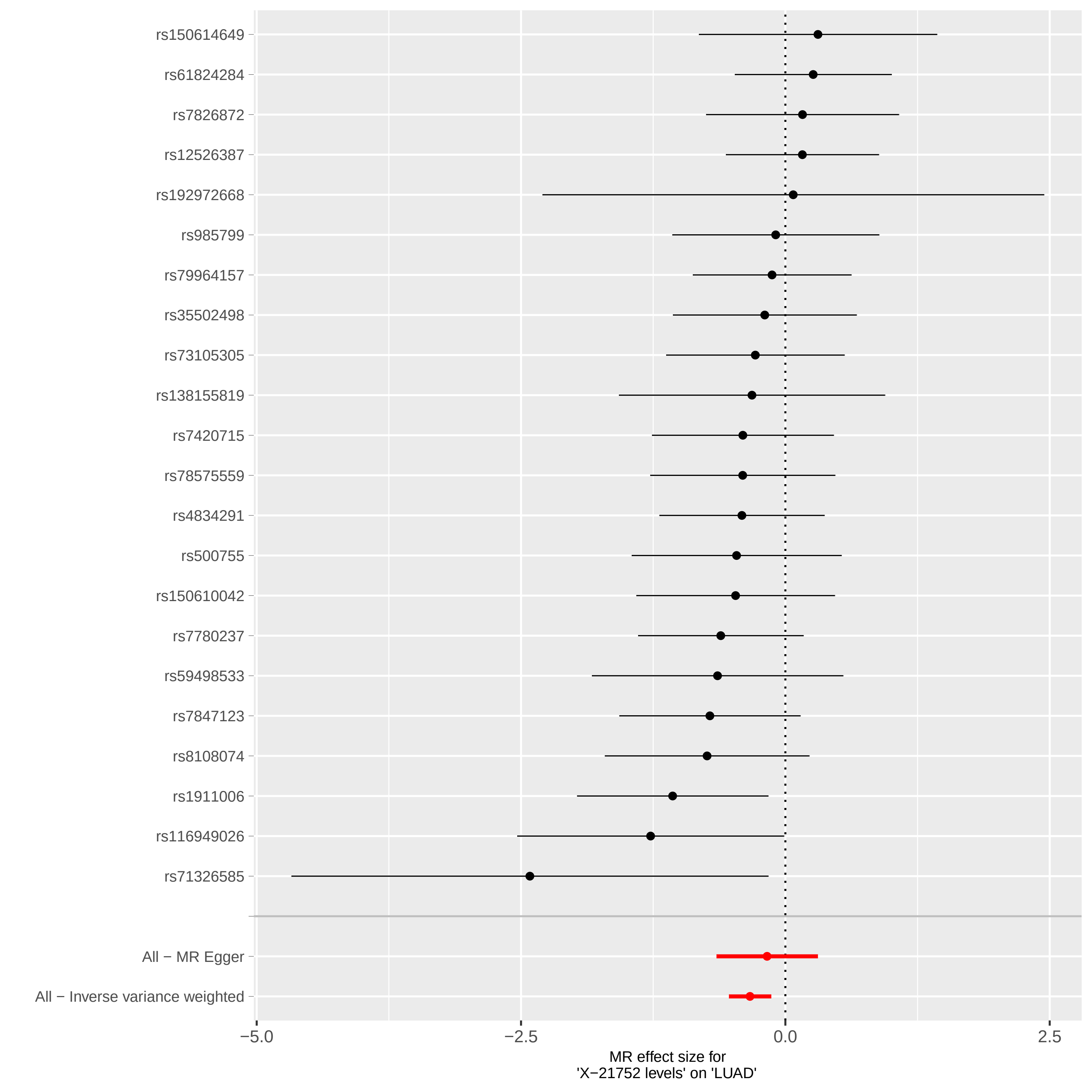
rs150614649
rs61824284
rs7826872
rs12526387
rs192972668
rs985799
rs79964157
rs35502498
rs73105305
rs138155819
rs7420715
rs78575559
rs4834291
rs500755
rs150610042
rs7780237
rs59498533
rs7847123
rs8108074
rs1911006
rs116949026
rs71326585
All − MR Egger
All − Inverse variance weighted
−5.0
−2.5
0.0
2.5
MR effect size for
'X−21752 levels' on 'LUAD'
